# Single-Cell Transcriptional Profiling of Cells Derived From Regenerating Alveolar Ducts

**DOI:** 10.3389/fmed.2020.00112

**Published:** 2020-04-21

**Authors:** Alexandra B. Ysasi, Robert D. Bennett, Willi Wagner, Cristian D. Valenzuela, Andrew B. Servais, Akira Tsuda, Saumyadipta Pyne, Shuqiang Li, Jonna Grimsby, Prapti Pokharel, Kenneth J. Livak, Maximilian Ackermann, Paul C. Blainey, Steven J. Mentzer

**Affiliations:** ^1^Laboratory of Adaptive and Regenerative Biology, Harvard Medical School, Brigham & Women's Hospital, Boston, MA, United States; ^2^Institute of Functional and Clinical Anatomy, University Medical Center of the Johannes Gutenberg-University, Mainz, Germany; ^3^Molecular and Integrative Physiological Sciences, Harvard School of Public Health, Boston, MA, United States; ^4^Public Health Dynamics Laboratory, University of Pittsburgh, Pittsburgh, PA, United States; ^5^Fluidigm Corporation, South San Francisco, CA, United States; ^6^Broad Institute of Harvard and MIT, Cambridge, MA, United States; ^7^Department of Biological Engineering, MIT, Cambridge, MA, United States

**Keywords:** warburg effect, glucose metabolism, aerobic glycolysis, metabolic reprogramming, cholangiocarcinoma

## Abstract

Lung regeneration occurs in a variety of adult mammals after surgical removal of one lung (pneumonectomy). Previous studies of murine post-pneumonectomy lung growth have identified regenerative “hotspots” in subpleural alveolar ducts; however, the cell-types participating in this process remain unclear. To identify the single cells participating in post-pneumonectomy lung growth, we used laser microdissection, enzymatic digestion and microfluidic isolation. Single-cell transcriptional analysis of the murine alveolar duct cells was performed using the C1 integrated fluidic circuit (Fluidigm) and a custom PCR panel designed for lung growth and repair genes. The multi-dimensional data set was analyzed using visualization software based on the tSNE algorithm. The analysis identified 6 cell clusters; 1 cell cluster was present only after pneumonectomy. This post-pneumonectomy cluster was significantly less transcriptionally active than 3 other clusters and may represent a transitional cell population. A provisional cluster identity for 4 of the 6 cell clusters was obtained by embedding bulk transcriptional data into the tSNE analysis. The transcriptional pattern of the 6 clusters was further analyzed for genes associated with lung repair, matrix production, and angiogenesis. The data demonstrated that multiple cell-types (clusters) transcribed genes linked to these basic functions. We conclude that the coordinated gene expression across multiple cell clusters is likely a response to a shared regenerative microenvironment within the subpleural alveolar ducts.

## Introduction

Lung regeneration occurs in a variety of adult mammals (1), including humans (2), after the surgical removal of one lung (pneumonectomy). The expansion of the remaining lung is not simple isotropic expansion as there is a commensurate increase in lung weight (3), cell number (4), and alveolar number (5). These observations indicate that post-pneumonectomy lung growth involves fundamental remodeling of the lung microarchitecture (6, 7).

The anatomic location of the compensatory remodeling appears to be in the peripheral alveolar ducts. Finite element modeling has shown that mechanical stretch—the signal most commonly implicated as a trigger in compensatory growth (1, 8)—is maximal in the subpleural alveolar ducts (9, 10). In many subpleural alveolar ducts, postpneumonectomy morphometry has demonstrated a retraction of alveolar septa and an increase in alveolar duct diameter (7). The time course of septal retraction suggests that ductal dilatation is followed by scattered cellular proliferation (11), the septal migration of myofibroblasts (12), and subsequent septal thickening (7). Ysasi et al. have speculated that these events participate in the repartitioning of the dilated alveolar ducts (7). Of note, histomorphometry has demonstrated no aggregates of proliferating cells, analogous to nonspecific inflammation or classic wound healing, within the remodeling alveolar duct septa (13).

Previous attempts to identify the cell-types participating in septal remodeling have used conventional cell isolation and cell labeling techniques. Transcriptional profiling of post-pneumonectomy lung cells, isolated by flow cytometry cell sorting, have facilitated the bulk analysis of endothelial cells (4, 14), monocytes (13), alveolar macrophages (15), type I and type II cells (16). These studies have demonstrated a variety of transcriptional profiles consistent with generalized growth, but no profile identifying a controlling cell-type. More recently, Lechner and colleagues have shown that blood-derived CCR2^+^ monocytes are important in modulating post-pneumonectomy lung growth (17).

To more comprehensively assess the cell-types and functional activities of cells within regenerative “hotspots” in the post-pneumonectomy cardiac lobe, we used laser microdissection and single-cell transcriptional profiling to characterize gene expression in the subpleural alveolar ducts. Single-cell transcriptional profiling of all the cells in the peripheral alveolar ducts produced multidimensional data sets that provided an opportunity for alternative approaches to analysis and visualization. To facilitate interpretation of these data sets, we used a data visualization map based on t-distributed stochastic neighbor embedding (tSNE) (18). The tSNE maps were further analyzed for transcriptional activity relevant to regenerative processes such as lung repair, matrix remodeling, and angiogenesis.

## Methods

### Animals

Male mice, eight to ten week old wild type C57BL/6 (Jackson Laboratory, Bar Harbor, ME, USA) were anesthetized as previously described (19). The care of the animals was consistent with guidelines of the American Association for Accreditation of Laboratory Animal Care (Bethesda, MD, USA) and approved by our Institutional Animal Care and Use Committee.

### Pneumonectomy

Each animal undergoing pneumonectomy was ventilated on a Flexivent (SciReq, Montreal, QC Canada) at ventilator settings of 200/min, 10 ml/kg, and PEEP of 2 cmH_2_O with a pressure limited constant flow profile (19). A left fifth intercostal space thoracotomy provided exposure for hilar ligation and left pneumonectomy. Postoperatively, the animal was weaned from mechanical ventilation and maintained on supplemental oxygen until normal spontaneous ventilation was observed. Plombage (20) and phrenic nerve (8) controls were performed as previously described and harvested on postoperative day 3.

### Euthanasia and Vascular Flushing

After the induction of anesthesia with intraperitoneal injection of ketamine 100 mg/kg (Fort Dodge Animal Health, Fort Dodge, IA, USA) and xylazine 10 mg/kg (Phoenix Scientific, St. Joseph, MO, USA), the animal was endotracheally intubated with an 18G angiocatheter (Becton Dickinson, Franklin Lakes, NJ, USA) and ventilated with the Flexivent rodent ventilator (SciReq, Montreal, Quebec, CA). The animal was euthanized by exsanguination through the inferior vena cava. A median sternotomy facilitated exposure of the anterior mediastinum. In sequence, the left atrium, right ventricle and inferior vena cava were incised. A 22G olive-tipped cannula was inserted through the right ventricle into the pulmonary artery and the lungs were flushed with 20 cc of phosphate-buffered saline at 23^o^C. A cervical tracheotomy was performed and the orotracheal tube replaced with a second 18G angiocatheter positioned in the distal trachea and secured with a silk tie. The lungs were inflated to 70% total lung capacity (TLC, based on the average of the volumes previously recorded on the Flexivent). During 70% TLC static inflation, the pulmonary artery was flushed first with 20cc of phosphate-buffered saline at 23^o^C prior preparation for precision-cut lung slices.

### Precision-Cut Lung Slices

Agarose at 3% (wt/vol) or alginate (1% wt/vol) and gelatin (5% wt/vol) were thoroughly mixed and warmed to 37°C. The trachea was cannulated and the warm embedding medium was infused through the trachea using the lowest pressure necessary to inflate the peripheral lung. At total lung capacity, the trachea was clamped and the lung block placed in 34 mM calcium chloride solution (in deionized water reconstituted to isotonicity with NaCl) at 4°C for 30 min to allow for gelation. Sectioning was performed with the Leica VT1000 S vibrating blade microtome (Leica Biosystems, Nussloch, Germany) using stainless steel razor blades (Gillette, Boston, MA). The microtome was operated at the following adjustable settings: knife angle, 5–7°; sectioning speed, 0.05– 0.2 mm/s; oscillation frequency, 80–100 Hz; and oscillation amplitude, 0.6 mm. Sections 200–300 μm thick were mounted on a polyethylene naphthalene membrane frame slide (Life Technologies) for laser microdissection.

### Laser Microdissection

The Arcturus XT LCM System (Life Technologies) was used for all ultraviolet (UV) laser dissection of precision-cut lung slices as previously described (21). The UV laser was specially adapted for wet tissue applications. The Arcturus XT software was used to target tissue for UV dissection. Peripheral alveolar ducts were identified in whole mounts as a minimum of six contiguous subpleural alveoli surrounding a central air space. Areas with ectopic anatomy were excluded from the analysis. Laser microdissection harvested alveolar ducts distal to the columnar-squamoid transition (21).

### Enzymatic Digestion

Enzymatic digestion of the lung reflected a previously published protocol (14). Briefly, 1 mg/ml collagenase Type IV (Worthington, Lakewood, NJ) and 0.01 mg/ml DNase I (Fisher Scientific, Pittsburgh, PA) in Dulbecco's modified Eagle's medium (Thermo Fisher Scientific, Waltham, MA) was used to dissociate the tissue. Dissociation was performed at 37°C under constant agitation for 30–45 min. The digest was filtered through 35 μm nylon mesh, and remaining debris was removed by centrifugation at 1,200 rpm for 3 min. The process of microfiltration and centrifugation was then repeated once more in preparation for microfluidic analysis.

### Viability Assessment

A small aliquot of cell suspension (7.5–10 μl) was used to assess cell concentration and viability by Trypan blue exclusion. Trypan blue (Sigma-Aldrich) was added in 1:1 ratio and cell concentration was determined using a standard microscope hematocytometer. Each cell counted was determined to be alive or dead based on Trypan blue exclusion and viability was calculated as a percentage of total cells.

### C1-Specific Target Amplification

Single mouse lung cell capture and STA (specific target amplification) were carried out using the Fluidigm C1 Single-Cell Auto Prep System and Single-Cell Auto Prep Array integrated fluidic circuits (IFCs) (Fluidigm, South San Francisco, CA). For these experiments, medium-sized (10–17 um cell diameter) STA IFCs were used. Chip-priming, cell-loading, lysis, reverse transcription, and preamplification were performed in accordance with Fluidigm's recommended protocol using lysis and preamplification reagents from the Single Cell-to-CT Kit (Ambion/Life Technologies) and pooled preamplification primers custom designed to enrich for 96 loci of interest (20). Cells were loaded onto the chip at concentrations of 120–370 cells/μl. After capture of individual cells on the C1 chip, the isolated cells were examined by light microscopy: 261 sites were excluded because of multiple cells (258 doublets; 103 triplets) and 347 were excluded because of associated cellular debris. The remaining single cells analyzed by the C1 chip included 1107 total single cells: 207 littermate control, 169 surgical controls (including postoperative day 3 plombage and phrenic nerve controls), 265 day 1, 261 day 3, 205 day 7 cells. Cell yields varied by timepoints suggesting a changing extracellular matrix; 23 mice were studied.

### Single-Cell Multiplexed Quantitative PCR

Preamplified cDNA samples from single cells were analyzed by qPCR using 96.96 Dynamic Array IFCs and the Biomark HD System from Fluidigm. Processing of the IFCs and operation of the instruments were performed according to the manufacturer's procedures and as previously described in detail (20). The performance of the qPCR was confirmed by systematic quality control analysis using the Bioanalyzer (Agilent, Santa Clara, CA, USA); results of the qPCR were consistent with previous findings (22). The custom gene panel was crowdsourced by a panel of 10 investigators involved in lung regeneration. The panel was selected by rank ordering genes-of-interest.

### tSNE

tSNE maps are a computational tool that permits the visualization of high-dimensional data sets in 2D maps using the tSNE algorithm (18). The data represented the Ct values of the qPCR data. The tSNE algorithm represents the distance between any 2 cells by the probability of these cells being neighbors; this spatial data is then represented in low dimensional space as a 2D map. We applied tSNE to compute the two-dimensional embedding of single-cell gene expression using the Cytobank viSNE software (Cytobank, Santa Clara, CA). tSNE was run using default parameters (iterations = 2000, perplexity = 10, theta = 0.5). In each figure, all samples were derived from the same tSNE run. tSNE heat maps show gene expression for each gene. Scales for each gene were individually generated from low to high expression and displayed on the right Y-axis. The SPADE cluster parameter permitted color overlay of the populations in the Cytobank working illustration.

### R Software

R software was used to perform a similarity analysis to compare the tSNE generated single cell clusters to transcriptional phenotypes of comparable cell populations analyzed by bulk flow cytometry. Combined single cell and cell-sorted bulk data were analyzed by R software in CSV file format. A subset of genes with variable expression profiles (*Col18a1, Col4a3, Csf3, Ctgf, Eng, Ephb4, Ereg, Fgf1, Igf1, Lep, Mapk14, Npr1, Pecam1, S1pr1, Sphk1, Tgfa, Tnf, Vegfa*) for 1107 single cell samples and 73 cell sorted bulk samples were the primary input for the matching algorithm. The R analysis script used gplots (23) and RColorBrewer (24) library packages mirrored through an R server in Berkeley, CA. A similarity matrix was generated with bulk and individual samples clustered by similarity based on the overlapping genes. Spatial coordinates of matching cells permitted projection of the bulk sample onto the tSNE two-dimensional dot plot (tSNE map).

### Data Analysis and Graphical Display

Data was analyzed with the Fluidigm Real-Time PCR Analysis software; the linear (derivative) baseline correction method and the auto (global) Ct threshold method was applied to determine expression. The Ct values determined were exported to Excel for further Processing using the Singular Analysis Toolset (Fluidigm) in the R software environment. The unpaired Student's *t*-test for samples of unequal variances was used to calculate statistical significance. The data was expressed as mean ± one standard deviation. The significance level for the sample distribution was defined as *p* < 0.05.

## Results

### Single-Cells From Alveolar Ducts

**Figure 1:**
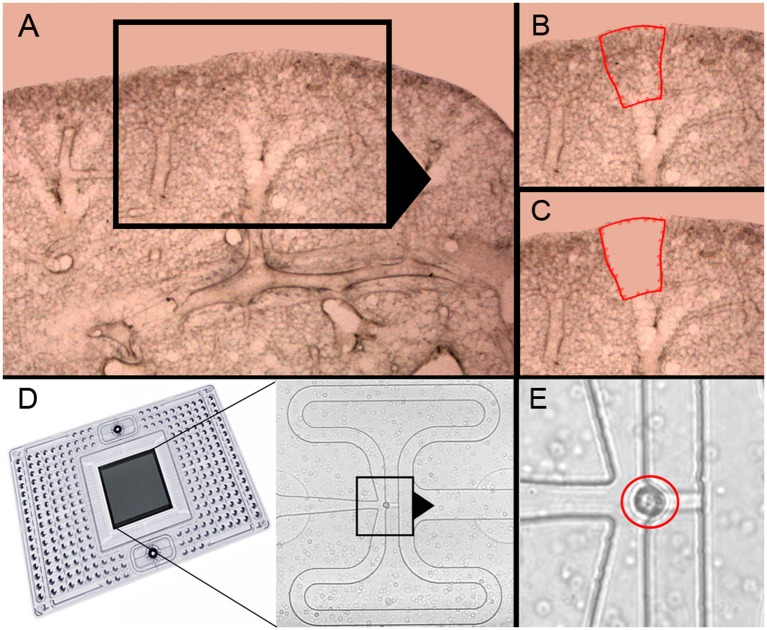
Precision-cut lung slices of the cardiac lobe, laser microdissection and microfluidic single-cell isolation. **(A–C)** The precision-cult lung slices (200 μm thick) examined at 10x and 20x magnification without counterstain. Alveolar ducts in the posterior curvature of the cardiac lobe were harvested by laser microdissection (21). **(D)** After enzymatic digestion and filtering, the cells were isolated on the C1 chip (Fluidigm). **(E)** Capture of individual cells without debris was confirmed by light microscopy (red circle).

Previous studies of post-pneumonectomy lung growth have identified regenerative “hotspots” in subpleural alveolar ducts (10) and in the posterior curvature of the cardiac lobe (12) (Figures 1A–C). To isolate single cells from these alveolar ducts, we used laser microdissection followed by enzymatic digestion (21). In 23 experiments, the average number of cells harvested by laser microdissection was 2.5 × 10^4^ ± 1.2 × 10^4^. The viability of the cells was 96 ± 3% by trypan blue exclusion. The final cell concentration was adjusted to optimize capture frequency prior to microfluidic isolation (Figure 1D). The mean cell capture frequency was 72%; 17% of the cells were excluded because cellular debris was associated with the isolated cells. Single-cells captured by the chip were confirmed by light microscopy prior to PCR (Figure 1E). These isolated single-cells were processed for gene expression using a crowdsourced custom panel of 96 genes selected for their association with lung growth. Cells were harvested from mice on post-pneumonectomy days 1, 3, and 7 as well as from littermate controls.

## Unclustered Transcription Pre-and Post-Pneumonectomy

**Figure 2:**
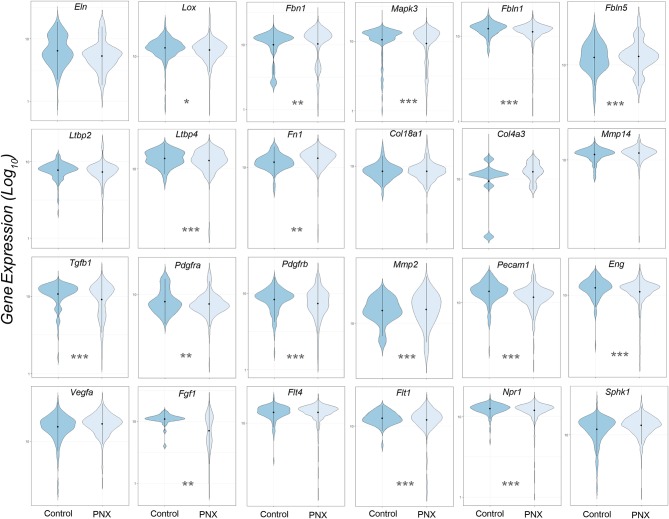
Violin plot comparison of gene transcription pre- and post-pneumonectomy. The transcription profiles of cells derived from littermate controls were compared to profiles obtained from post-pneumonectomy (PNX) mice in the first week after surgery. The data for 24 genes linked to lung repair, matrix production and angiogenesis are shown. Gene expression is shown as log_10_. Student's test level of significance: *p < 0.05, **p < 0.01, ***p < 0.001.

The transcriptional profiles of individual genes for cells obtained from littermate controls was compared to the aggregate of cells obtained post-pneumonectomy (Figure 2). Analogous to previous studies using bulk analyses, differences in gene expression were observed in most genes, but the biological significance was unclear.

## Cell Cluster Identity

**Figure 3:**
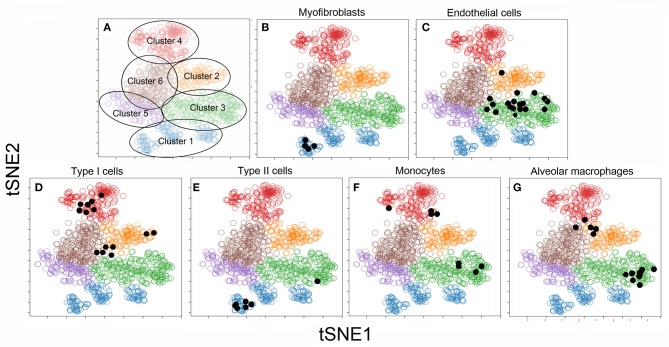
tSNE clustering of the combined single-cell transcriptional data (colored circles) and embedded bulk transcriptional data (black dots) in 2D maps. The analysis was statistically constrained to 6 clusters. The 6 clusters were color-coded for presentation purposes **(A)**. To obtain a provisional cell-type identity for the clusters, previously obtained post-pneumonectomy bulk transcriptional data were embedded into the tSNE analyses. The results of these embedded analyses were projected on the tSNE map (shown as black dots) for 6 cell-types. Note, the bulk transcriptional data is typically not restricted to one cluster, but may be linked to multiple clusters. For this reason, the cell-type identities are considered provisional: Cluster 1: α-smooth muscle actin myofibroblasts (20) **(B)** and Type II cells (16) **(E)**; Cluster 2: CD11b^+^ monocytes (13) **(F)** and F4/80^+^ alveolar macrophages (15) **(G)**; Cluster 3: CD31^+^ endothelial cells (4) **(C)**; Cluster 4: CD11b^−^/CD31^−^ epithelial Type I cells (16) **(D)**. There was no provisional identity for Clusters 5 and 6.

To facilitate visual processing of the single-cell data set, we used tSNE and SPADE software to plot 6 color-coded clusters (Figure 3A). The clusters reflect the similarities of the individual cells in high-dimensional space using the tSNE algorithm. To infer the conventional cell identities within the 6 clusters, we used raw data from previously published bulk analyses. A matching algorithm, based on 36 overlapping genes, was used to project the results of the bulk data onto the tSNE plots. Using this approach, Cluster 1 was the projection of myofibroblasts (20) (Figure 3B), Type II cells (16) (Figure 3E), and endothelial progenitor cells (14) (not shown). Cluster 2, notable for the dramatic increase in number after pneumonectomy, was a poorly defined regenerative cell population partly representing alveolar macrophages (15) (Figure 3G). Cluster 3 was the projection of endothelial cells defined by cell sorting on the CD31 cell surface molecule (4) (Figure 3C). Cluster 4 reflected epithelial Type I cells (16) (Figure 3D) and monocytes defined by cell sorting on the CD11b cell surface molecule (13) (Figure 3F).

**Figure 4:**
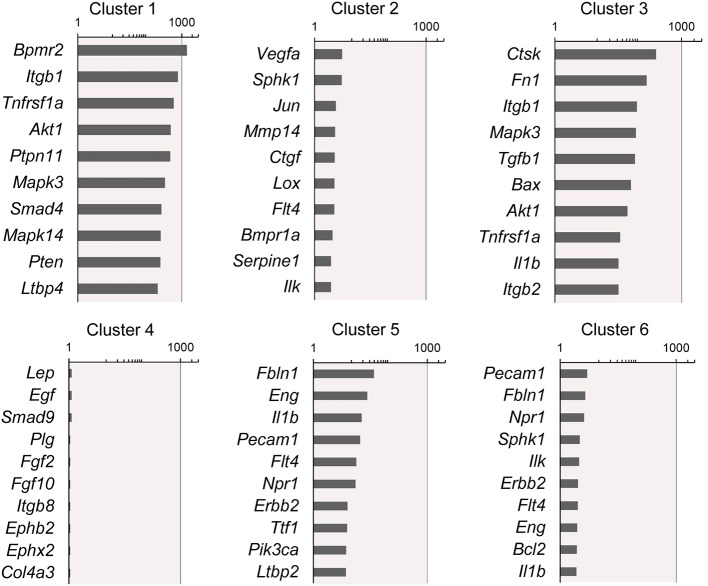
Rank-order of highest gene expression within each cluster. The log_2_ fold-change in gene expression of the 10 highest expressing genes in each cluster compared to the remaining 5 clusters. The data from all timepoints are shown. The remainder of the 86 genes are presented in Supplementary Data.

### Cluster Changes After Pneumonectomy

**Figure 5:**
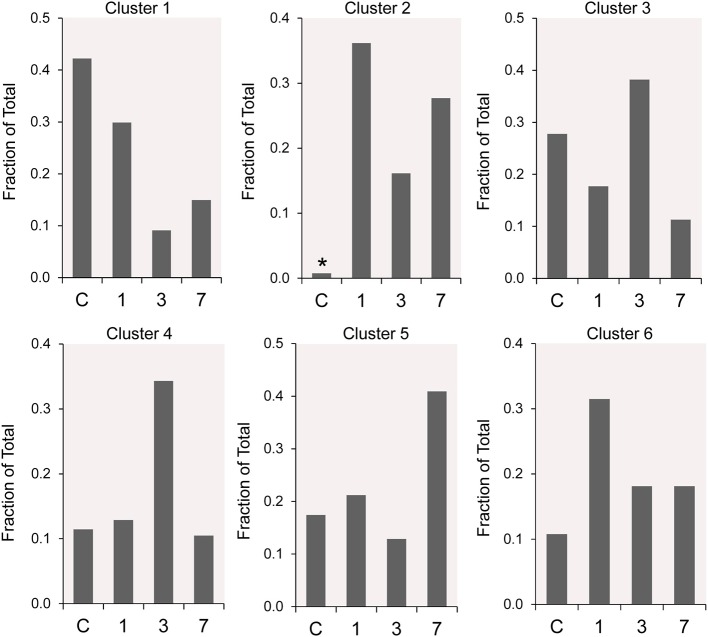
The time course distribution of cluster frequency s analyzed by single-cell qPCR. The single-cells in each cluster are shown as a fraction of the total number of cells isolated at each time point. The single-cells isolated from littermate controls (C) were compared to cells isolated from regenerative alveolar ducts on postoperative day 1 (20), day 3 (11), and day 7 (13). Plombage and phrenic nerve controls are not shown. Note the low frequency of Cluster 2 in littermate controls (*).

The frequency distribution of the 6 clusters demonstrated changes over the 7 days post-pneumonectomy (Figure 5). Most notably, Cluster 2, virtually unrepresented in control conditions, was prominent in all three timepoints studied post-pneumonectomy (Figure 5, asterisk). Littermate control and postoperative day 3 plombage and phrenic nerve transection were indistinguishable.

### Clusters and Functional Associations

**Figure 6:**
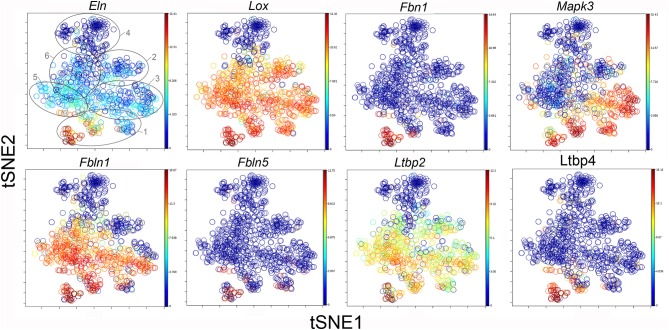
TSNE map of the combined single-cell transcription of genes classified as lung repair genes. The map is color-coded to reflect relative gene expression. The genes are elastin (*Eln*), lysyl oxidase (*Lox*), fibrillin (*Fbn1*), mitogen-activated protein kinase 3 (*Mapk3*), fibulin 1 (*Fbln1*), fibulin 5 (*Fbln5*), latent-transforming growth factor beta-binding protein 2 (*Ltbp2*) and 4 (*Ltbp4*). The linked pattern of gene expression is apparent for *Eln, Lox*, and *Ltbp2*. Cluster 1 expression of the lung repair genes is high for all lung repair genes. All timepoints are combined in the analysis. In the first panel, the cluster boundaries are shown for clarity.

**Figure 7:**
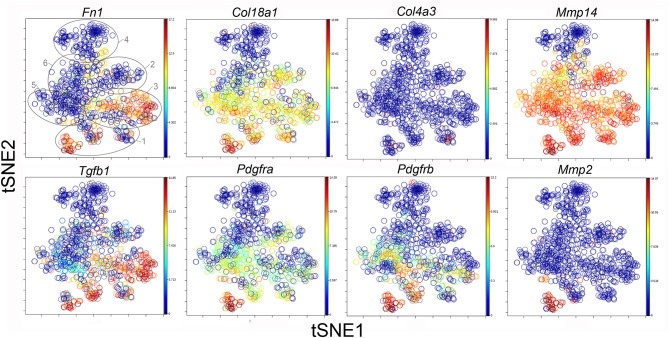
The tSNE map of the combined single-cell transcription of genes classified as lung matrix genes. The map is color-coded to reflect relative gene expression. The genes are fibronectin-1 (*Fn1*), collagen Type XV111 alpha-1 (*Col18a1*), collagen Type IV alpha-3 (*Col4a3*), matrix metalloproteinase 2 (*Mmp2*) and 14 (*Mmp14*), transforming growth factor beta-1 (*Tgfb1*), platelet-derived growth factor receptor alpha (*Pdgfra*) and beta (*Pdgfrb*). The linked pattern of gene expression is apparent for *Col18a1* and *Mmp14* as well as *Fn1* and *Tgfb1*. Cluster 1 expression of the lung matrix genes is high, but variable within the Cluster 1 subpopulations. All timepoints are combined in the analysis. In the first panel, the cluster boundaries are shown for clarity.

**Figure 8:**
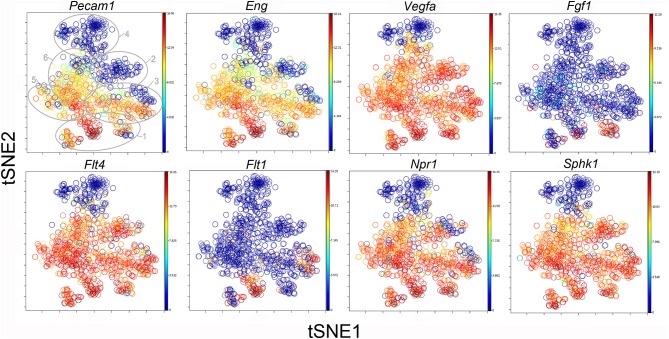
The tSNE map of the combined single-cell transcription of genes classified as angiogenesis genes. The map is color-coded to reflect relative gene expression. The genes areH platelet endothelial cell adhesion molecule 1 (*Pecam1*), endoglin (*Eng*), vascular endothelial growth factor A (*Vegfa*), fibroblast growth factor 1 (*Fgf1*), fms-related tyrosine kinase receptor 1 (*Flt1*) and 4 (*Flt4*), naturetic peptide receptor 1 (*Npr1*) and sphingosine kinase 1 (*Sphk1*). The linked pattern of gene expression is apparent for *Pecam1* and *Eng* as well as *Vegfa, Flt4, Npr1*, and *Sphk1*. Almost all subpopulations of Cluster 1 demonstrate high expression of angiogenesis genes. All timepoints are combined in the analysis. In the first panel, the cluster boundaries are shown for clarity.

The participation of the six clusters in post-pneumonectomy lung growth was explored by examining genes linked to (20) lung repair, (21) matrix production, and (11) angiogenesis. When lung repair genes were analyzed (Figure 5), a prominent finding was the high level of transcriptional activity in Cluster 1 (see Supplementary Data). The 3 subpopulations within Cluster 1 (myofibroblasts, Type II cells and CD34^+^ endothelial progenitors) indicated under-clustering of these cell populations. An interesting observation was the varied multi-cluster pattern of transcription associated with function-associated genes. Notably, elastin (*Eln*), the elastin crosslinking enzyme lysyl oxidase (*Lox*) and the elastin scaffold protein fibrillin1 (*Fbn1*) demonstrated a similar pattern of expression in Cluster 1, but variable expression in multiple clusters (Figure 5). The production of lung extracellular matrix was similarly evaluated (Figure 6). In addition to the prominent transcriptional activity of Cluster 1, Cluster 3 demonstrated prominent expression of matrix proteins such as fibronectin (*Fn1*), as well as mediators (*Tgfb1*) and regulatory kinases (*Mapk3*) (see Supplementary Data). Notably, the multi-cluster pattern of collagen (*Col18a*) and matrix proteases (*Mmp14*) transcription suggested common gene regulation. The expression of angiogenesis-related genes appeared to involve all clusters with the exception of Cluster 4 (Figure 7). The similar expression pattern of these angiogenesis genes was notable in the plots of *Vegfa, Flt4, Npr1* and *Sphk1*. In contrast to many structural genes, angiogenesis genes were broadly expressed in the cell population defined by Cluster 2 (Figure 8).

## Discussion

In this report, our objective was to define the transcriptional activity of single-cells isolated from alveolar ducts located in anatomic regions considered to be regenerative “hot spots” of lung growth after pneumonectomy. Laser microdissection facilitated the isolation of alveolar duct cells without the contamination of central airway cells or the confounding influence of inflammatory cells. The single-cell transcriptional activity was assessed using a crowdsourced panel of genes selected for their relevance to lung growth. The multidimensional data, clustered and mapped using the tSNE algorithm, demonstrated coordinated gene expression across multiple clusters. We interpret these findings as reflecting a common response to a shared regenerative microenvironment.

An interesting finding of these studies was challenge of identifying the correspondence between tSNE clusters and conventional cell-types. Classifying cells based on their properties is the conventional basis for reasoning about biologic processes and cell function (25, 26). Light and electron microscopy, demonstrating cell size, shape and ultrastructural features, have led to classifications based on cell morphotypes (27). Cell surface molecule expression, often defined by antibodies, can result in cell classification based on molecular phenotype (28, 29). More recently, single cell transcriptional profiling provides an opportunity to classify cells based on gene expression. Progress with single-cell transcriptional profiling, however has been limited by large multidimensional data sets often resistant to straightforward classification using canonical markers or landmark genes.

To address these limitations of multidimensional transcriptional data, we used a computational technique of dimensionality reduction called tSNE (18), a variation of stochastic neighbor embedding (30). tSNE is a nonlinear data reduction tool that permits the high-dimensional similarities of cells to be visualized in a 2-dimensional scatter plot. The spatial relationship of cells in the tSNE map reflects their distances in high-dimensional space; that is, cells with similar gene expression patterns are clustered together in the tSNE map. The tSNE map has several advantages for application to lung repair and regeneration. tSNE is an unsupervised algorithm that does not require prior knowledge or assumptions about cellular states. Moreover, tSNE reduces the complexity of the presentation, but preserves the resolution—all individual cells are plotted in the tSNE map.

To facilitate classification of the tSNE clusters, our approach was to embed previously obtained conventional bulk transcriptional analyses onto the tSNE algorithm. Our conventional analyses used flow cytometry cell sorting to isolate canonically defined cell-types prior to bulk transcriptional profiling. For example, flow cytometry-based cell sorting of CD31^+^ cells produced the transcriptional profiles of conventionally-defined endothelial cells. By embedding the bulk data into the algorithm and projecting these data onto the tSNE map, we were able to provide a spatial reference for CD31-defined endothelial cells in Cluster 3. The relevance of this assumption is supported by the significant angiogenesis-related transcriptional activity of the cells in Cluster 3. We caution, however, that the genes analyzed in the bulk analyses and single-cell studies were not identical (typically 36 overlapping genes). Moreover, there are inherent problems in normalizing different data sets. The inferences based on the bulk data are best viewed as hypotheses or provisional data projections to be investigated in future studies.

An intriguing cell population, demonstrating persistently high transcriptional activity, was identified by Cluster 1. Previous work has identified alpha-smooth muscle actin (*Acta2*^+^) myofibroblasts in this population (20). A similar transcriptional profile, but without the expression of the alpha-smooth muscle gene (*Acta2*), suggested the presence of related fibroblast populations. An unexpected finding was the presence of two other conventionally-defined cell populations within Cluster I. Bulk analysis of Type II cells, based on phospholipid expression (16) as well as the bulk analysis of CD34^+^ cells (14) also projected within this cluster. Our data indicates that the low frequency of these cells will require a significantly larger sample size to improve the resolution of this cluster; nonetheless, our data suggests that Cluster 1 reflects highly active cells that regulate a variety of functions during lung repair and regeneration. We speculate that the profound effect of the absence of some genes, such as Pdgfra (31), on alveolarization reflects the relatively restricted expression of these genes relative to other cell clusters.

For most cell clusters, the tSNE maps remained relatively stable both pre- and post-pneumonectomy. The exception was Cluster 2. Prior to pneumonectomy, fewer than 1 percent of the cells were represented in Cluster 2; in the 7 days after pneumonectomy, a mean of 27% of the cells were defined by Cluster 2. The Cluster 2 population demonstrated transcriptional activity in a variety of functions related to lung repair and angiogenesis. The varied transcriptional activity of this cluster suggests that Cluster 2 represents a transitional population—perhaps reflecting basic cellular processes during lung repair (e.g., cell cycling). Also, consistent with recent reports (17), monocytes and alveolar macrophages projected in Cluster 2. Cluster 2 is an intriguing population that deserves future study.

Finally, our data indicate the need to modify conventional notions that a single gene is exclusively linked to a cell-type, or a single cell-type controls a corresponding biologic process. Our data suggests that genes previously considered canonical markers can be expressed in multiple cells types; similarly, multiple cell clusters can participate in basic biologic processes such as angiogenesis, lung repair and parenchymal remodeling. No single cell-type alone—at least none of those cell-types identified by multidimensional transcriptional profiling—controlled these processes. Our data suggests that multiple cell-types (clusters) can transcribe genes linked to a given function. We suspect this coordinated gene expression is a response to shared microenvironmental cues. Defining the nature of these signals may provide an opportunity to stimulate or inhibit lung growth.

## Data Availability Statement

All datasets generated for this study are included in the article/Supplementary Material.

## Ethics Statement

The animal care approval was obtained from the Brigham & Women & Hospital IACUC. The studies were carried out in accordance with the recommendations of the American Association for Accreditation of Laboratory Animal Care.

## Author Contributions

AY, RB, WW, CV, and AS performed the pneumonectomy and laser microdissection. SP, SL, JG, PP, and KL performed and supervised single-cell analysis and interpretation. AY, AT, MA, PB, and SM performed data interpretation and manuscript preparation.

## Conflict of Interest

SL, JG, PP, and KL were employees of the Fluidigm Corporation. The remaining authors declare that the research was conducted in the absence of any commercial or financial relationships that could be construed as a potential conflict of interest.

## Supplementary Material

Supplementary Data Supplementary data includes single-cell transcriptional data by cluster (**Data Sheets 1–3**) and post-pneumonectomy day (**Data Sheets 4–7**).

## Abbreviations

PNX: pneumonectomy
tSNE: t-distributed stochastic neighbor embedding
TLC: total lung capacity.
