# Supplementary material for: Single-Cell Transcriptional Profiling of Cells Derived From Regenerating Alveolar Ducts
Source: Front Med (Lausanne). 2020 Apr 21;7:112. doi: 10.3389/fmed.2020.00112 (PMC7186418; doi:10.3389/fmed.2020.00112)
Supplement: Supplementary file 1 [file Data_Sheet_1.PDF]

Cytobank

Experiment	single cell histograms
File	Steven's Working Illustration
Created/Updated	2017-10-31 13:20:43 -0700 / 2017-11-09 11:55:30 -0800
Illustration Author	Steven Mentzer
Primary Researcher	Steven Mentzer

Steven's Working Illustration

Histogram Overlay

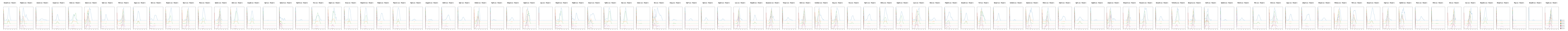

Calculated Fold of Means by First Row using X-Axis channel(s): Panel/Channel Values

	Smad4 (v) - Panel 1	Pdgfra (v) - Panel 1	Acta2 (v) - Panel 1	Casp3 (v) - Panel 1	Ctsk (v) - Panel 1	Ephx1 (v) - Panel 1	Fgf1 (v) - Panel 1	Flt4 (v) - Panel 1	Itgav (v) - Panel 1	Kdr (v) - Panel 1	Mapk1 (v) - Panel 1	Npr1 (v) - Panel 1	Pten (v) - Panel 1	Sphk1 (v) - Panel 1	Akt1 (v) - Panel 1	Casp8 (v) - Panel 1	Egf (v) - Panel 1	Ephx2 (v) - Panel 1
cluster 1	1.0	1.0	1.0	1.0	1.0	1.0	1.0	1.0	1.0	1.0	1.0	1.0	1.0	1.0	1.0	1.0	1.0	1.0
cluster 2	0.12	0.48	0.12	0.11	0.38	0.6	0.1	0.9	0.17	0.04	0.14	0.61	0.09	0.95	0.16	0.09	0.0	0.09
cluster 3	0.46	0.48	0.09	0.59	1.51	0.67	0.09	0.95	0.45	0.13	0.69	0.83	0.59	0.92	0.73	0.82	0.5	0.65
cluster 4	0.01	0.03	0.07	0.03	0.14	0.06	0.0	0.09	0.01	0.09	0.02	0.06	0.02	0.07	0.04	0.03	0.0	0.53
cluster 5	0.14	0.68	0.08	0.19	0.21	0.75	0.17	1.04	0.07	0.26	0.11	0.94	0.13	0.98	0.09	0.08	0.24	0.62
cluster 6	0.03	0.35	0.03	0.09	0.2	0.56	0.08	0.87	0.01	0.13	0.07	0.82	0.07	0.89	0.08	0.09	0.29	0.7

	Fgf10 (v) - Panel 1	Fn1 (v) - Panel 1	Itgb1 (v) - Panel 1	Kras (v) - Panel 1	Mapk14 (v) - Panel 1	Pdgfa (v) - Panel 1	Ptpn11 (v) - Panel 1	Tgfa (v) - Panel 1	Angpt2 (v) - Panel 1	Cdh5 (v) - Panel 1	Egfr (v) - Panel 1	Erbb2 (v) - Panel 1	Fgf2 (v) - Panel 1	Hbegf (v) - Panel 1	Itgb2 (v) - Panel 1	Lep (v) - Panel 1
cluster 1	1.0	1.0	1.0	1.0	1.0	1.0	1.0	1.0	1.0	1.0	1.0	1.0	1.0	1.0	1.0	1.0
cluster 2	0.0	0.22	0.3	0.2	0.13	0.08	0.12	0.15	0.37	0.04	0.04	0.47	0.0	0.19	0.78	0.0
cluster 3	0.07	1.26	0.76	0.52	0.69	0.09	0.45	0.02	0.19	0.12	0.07	0.74	0.01	0.08	1.18	0.0
cluster 4	0.05	0.12	0.08	0.03	0.03	0.01	0.05	0.04	0.0	0.11	0.0	0.1	0.04	0.02	0.11	0.43
cluster 5	0.05	0.17	0.19	0.1	0.1	0.13	0.1	0.09	0.31	0.24	0.06	0.91	0.04	0.06	0.97	0.0
cluster 6	0.04	0.16	0.08	0.04	0.08	0.06	0.07	0.04	0.12	0.08	0.03	0.77	0.04	0.02	0.8	0.0

	Mapk3 (v) - Panel 1	Pdgfb (v) - Panel 1	S1pr1 (v) - Panel 1	Tgfb1 (v) - Panel 1	Bax (v) - Panel 1	Cela1 (v) - Panel 1	Eln (v) - Panel 1	Ereg (v) - Panel 1	Fgf7 (v) - Panel 1	Hgf (v) - Panel 1	Itgb3 (v) - Panel 1	Lox (v) - Panel 1	Mapk8 (v) - Panel 1	Serpine1 (v) - Panel 1	Timp1 (v) - Panel 1	Bcl2 (v) - Panel 1
cluster 1	1.0	1.0	1.0	1.0	1.0	1.0	1.0	1.0	1.0	1.0	1.0	1.0	1.0	1.0	1.0	1.0
cluster 2	0.22	0.09	0.42	0.3	0.17	0.67	0.26	0.04	0.0	0.08	0.0	0.84	0.06	0.83	0.07	0.89
cluster 3	0.82	0.19	0.43	1.05	0.85	2.12	0.38	0.7	0.07	0.39	0.14	0.88	0.48	0.94	0.27	0.95
cluster 4	0.07	0.13	0.03	0.03	0.05	0.19	0.06	0.17	0.01	0.02	0.06	0.07	0.02	0.08	0.02	0.26
cluster 5	0.25	0.17	0.62	0.36	0.07	0.12	0.59	0.1	0.08	0.06	0.32	0.92	0.08	0.92	0.08	0.94
cluster 6	0.17	0.1	0.29	0.18	0.08	0.38	0.36	0.04	0.01	0.05	0.01	0.74	0.07	0.68	0.01	0.9

	Col18a1 (v) - Panel 1	Eng (v) - Panel 1	Fas (v) - Panel 1	Fgfr1 (v) - Panel 1	Hif1a (v) - Panel 1	Itgb5 (v) - Panel 1	Lrp1 (v) - Panel 1	Met (v) - Panel 1	Pdgfrb (v) - Panel 1	Smad2 (v) - Panel 1	Tnf (v) - Panel 1	Bmp4 (v) - Panel 1	Col4a3 (v) - Panel 1	Epha2 (v) - Panel 1	Fbln1 (v) - Panel 1
cluster 1	1.0	1.0	1.0	1.0	1.0	1.0	1.0	1.0	1.0	1.0	1.0	1.0	1.0	1.0	1.0
cluster 2	0.67	0.33	0.07	0.07	0.21	0.04	0.69	0.31	0.03	0.12	0.75	0.08	0.12	0.55	0.25
cluster 3	0.72	0.78	0.36	0.16	0.73	0.86	1.0	0.22	0.5	0.56	1.05	0.05	0.0	0.53	0.88
cluster 4	0.03	0.06	0.01	0.03	0.01	0.09	0.16	0.01	0.03	0.0	0.13	0.02	0.0	0.03	0.07
cluster 5	0.82	0.96	0.04	0.12	0.17	0.16	0.84	0.09	0.6	0.01	0.91	0.12	0.19	0.68	1.13
cluster 6	0.49	0.72	0.03	0.02	0.04	0.06	0.75	0.06	0.42	0.03	0.63	0.03	0.11	0.33	0.86

	Fgfr2 (v) - Panel 1	Igf1 (v) - Panel 1	Itgb8 (v) - Panel 1	Ltbp2 (v) - Panel 1	Mmp14 (v) - Panel 1	Pecam1 (v) - Panel 1	Smad3 (v) - Panel 1	Tnfrsf1a (v) - Panel 1	Bmpr1a (v) - Panel 1	Csf3 (v) - Panel 1	Ephb2 (v) - Panel 1	Fbln5 (v) - Panel 1	Flt1 (v) - Panel 1	Il1b (v) - Panel 1	Jag1 (v) - Panel 1	Ltbp4 (v) - Panel 1
cluster 1	1.0	1.0	1.0	1.0	1.0	1.0	1.0	1.0	1.0	1.0	1.0	1.0	1.0	1.0	1.0	1.0
cluster 2	0.22	0.07	0.0	0.69	0.85	0.06	0.11	0.18	0.9	0.32	0.0	0.02	0.01	0.17	0.11	0.01
cluster 3	0.12	0.67	0.11	0.82	0.88	0.73	0.14	0.68	0.95	0.88	0.03	0.04	0.43	1.12	0.13	0.07
cluster 4	0.04	0.02	0.01	0.04	0.11	0.06	0.01	0.07	0.13	0.05	0.0	0.03	0.0	0.11	0.03	0.05
cluster 5	0.18	0.08	0.0	0.98	0.91	0.89	0.1	0.22	0.96	1.12	0.05	0.17	0.08	1.11	0.06	0.18
cluster 6	0.1	0.06	0.01	0.72	0.78	0.74	0.02	0.05	0.86	0.79	0.0	0.0	0.03	0.85	0.03	0.03

	Mmp2 (v) - Panel 1	Pik3ca (v) - Panel 1	Ttf1 (v) - Panel 1	Bmpr2 (v) - Panel 1	Ctgf (v) - Panel 1	Ephb4 (v) - Panel 1	Fbn1 (v) - Panel 1	Flt3 (v) - Panel 1	Ilk (v) - Panel 1	Jun (v) - Panel 1	Map2k1 (v) - Panel 1	Mmp9 (v) - Panel 1	Plg (v) - Panel 1	Smad9 (v) - Panel 1	Vegfa (v) - Panel 1
cluster 1	1.0	1.0	1.0	1.0	1.0	1.0	1.0	1.0	1.0	1.0	1.0	1.0	1.0	1.0	1.0
cluster 2	0.04	0.68	0.66	0.08	0.86	0.51	0.04	0.19	0.89	0.84	0.07	0.94	0.18	0.0	0.95
cluster 3	0.04	0.8	0.74	0.37	0.87	0.59	0.04	1.19	0.95	0.86	0.65	1.21	0.45	0.0	0.91
cluster 4	0.02	0.01	0.12	0.03	0.05	0.03	0.02	0.0	0.22	0.23	0.01	0.21	0.0	0.0	0.23
cluster 5	0.17	0.83	0.97	0.12	0.94	0.79	0.1	0.15	0.95	0.82	0.08	3.69	1.18	0.0	0.92
cluster 6	0.02	0.51	0.45	0.04	0.75	0.36	0.02	0.12	0.89	0.74	0.04	1.02	0.5	0.0	0.84
