# Supplementary material for: Single-Cell Transcriptional Profiling of Cells Derived From Regenerating Alveolar Ducts
Source: Front Med (Lausanne). 2020 Apr 21;7:112. doi: 10.3389/fmed.2020.00112 (PMC7186418; doi:10.3389/fmed.2020.00112)
Supplement: Supplementary file 2 [file Data_Sheet_2.PDF]

Cytobank

Steven's Working Illustration

Experiment	single cell histograms
File	Steven's Working Illustration
Created/Updated	2017-10-31 13:20:43 -0700 / 2017-11-03 06:30:02 -0700
Illustration Author	Steven Mentzer
Primary Researcher	Steven Mentzer

Histogram Overlay

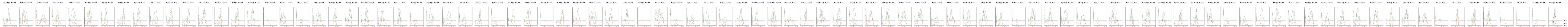

Calculated Fold of Means by First Row using X-Axis channel(s): Panel/Channel Values

	Smad4 (v) - Panel 1	Pdgfra (v) - Panel 1	Acta2 (v) - Panel 1	Casp3 (v) - Panel 1	Ctsk (v) - Panel 1	Ephx1 (v) - Panel 1	Fgf1 (v) - Panel 1	Flt4 (v) - Panel 1	Itgav (v) - Panel 1	Kdr (v) - Panel 1	Mapk1 (v) - Panel 1	Npr1 (v) - Panel 1
POD0	1.0	1.0	1.0	1.0	1.0	1.0	1.0	1.0	1.0	1.0	1.0	1.0
POD1	1.0	1.24	1.32	1.46	0.85	0.93	0.95	1.14	1.34	0.64	1.01	1.02
POD3	0.87	1.39	2.28	1.53	0.84	0.92	1.27	0.94	1.24	0.29	0.84	0.91
POD7	0.83	1.5	2.14	1.07	0.53	0.86	0.5	1.13	0.72	0.41	0.53	1.0

	Pten (v) - Panel 1	Sphk1 (v) - Panel 1	Akt1 (v) - Panel 1	Casp8 (v) - Panel 1	Egf (v) - Panel 1	Ephx2 (v) - Panel 1	Fgf10 (v) - Panel 1	Fn1 (v) - Panel 1	Itgb1 (v) - Panel 1	Kras (v) - Panel 1	Mapk14 (v) - Panel 1	Pdgfa (v) - Panel 1
POD0	1.0	1.0	1.0	1.0	1.0	1.0	1.0	1.0	1.0	1.0	1.0	1.0
POD1	1.07	1.22	1.16	0.85	0.77	0.52	0.61	0.86	1.06	1.16	1.43	1.52
POD3	0.96	1.01	1.07	0.66	0.0	0.41	1.1	1.22	1.0	0.97	1.19	1.63
POD7	0.73	1.19	0.98	0.3	0.72	0.92	2.05	1.33	0.94	0.75	1.06	1.1

	Ptpn11 (v) - Panel 1	Tgfa (v) - Panel 1	Angpt2 (v) - Panel 1	Cdh5 (v) - Panel 1	Egfr (v) - Panel 1	Erbb2 (v) - Panel 1	Fgf2 (v) - Panel 1	Hbegf (v) - Panel 1	Itgb2 (v) - Panel 1	Lep (v) - Panel 1	Mapk3 (v) - Panel 1	Pdgfb (v) - Panel 1
POD0	1.0	1.0	1.0	1.0	1.0	1.0	1.0	1.0	1.0	1.0	1.0	1.0
POD1	1.06	4.34	0.29	0.65	2.15	1.01	1.17	5.54	1.01	0.0	1.0	0.87
POD3	1.18	6.4	0.28	0.12	2.34	0.98	1.26	4.1	0.71	0.0	0.94	0.33
POD7	0.98	3.24	0.08	0.35	1.4	1.09	1.39	1.37	1.01	0.0	0.81	0.41

	S1pr1 (v) - Panel 1	Tgfb1 (v) - Panel 1	Bax (v) - Panel 1	Cela1 (v) - Panel 1	Eln (v) - Panel 1	Ereg (v) - Panel 1	Fgf7 (v) - Panel 1	Hgf (v) - Panel 1	Itgb3 (v) - Panel 1	Lox (v) - Panel 1	Mapk8 (v) - Panel 1	Serpine1 (v) - Panel 1
POD0	1.0	1.0	1.0	1.0	1.0	1.0	1.0	1.0	1.0	1.0	1.0	1.0
POD1	0.87	0.72	1.05	1.0	0.84	1.11	1.0	0.51	0.81	1.04	1.7	1.07
POD3	0.64	0.53	1.14	0.97	0.81	0.94	2.19	0.49	0.68	1.07	1.74	1.03
POD7	0.84	0.65	0.77	0.0	1.17	1.57	1.42	0.48	0.94	1.08	0.65	1.05

	Timp1 (v) - Panel 1	Bcl2 (v) - Panel 1	Col18a1 (v) - Panel 1	Eng (v) - Panel 1	Fas (v) - Panel 1	Fgfr1 (v) - Panel 1	Hif1a (v) - Panel 1	Itgb5 (v) - Panel 1	Lrp1 (v) - Panel 1	Met (v) - Panel 1	Pdgfrb (v) - Panel 1	Smad2 (v) - Panel 1
POD0	1.0	1.0	1.0	1.0	1.0	1.0	1.0	1.0	1.0	1.0	1.0	1.0
POD1	1.6	1.18	1.28	0.86	0.75	1.06	1.54	0.79	1.15	3.22	0.58	1.33
POD3	2.12	1.05	1.1	0.62	0.37	1.32	1.42	0.98	1.02	4.3	0.64	1.35
POD7	2.4	1.06	1.32	0.88	0.29	1.4	1.15	0.9	1.09	2.53	0.87	0.58

	Tnf (v) - Panel 1	Bmp4 (v) - Panel 1	Col4a3 (v) - Panel 1	Epha2 (v) - Panel 1	Fbln1 (v) - Panel 1	Fgfr2 (v) - Panel 1	Igf1 (v) - Panel 1	Itgb8 (v) - Panel 1	Ltbp2 (v) - Panel 1	Mmp14 (v) - Panel 1	Pecam1 (v) - Panel 1	Smad3 (v) - Panel 1
POD0	1.0	1.0	1.0	1.0	1.0	1.0	1.0	1.0	1.0	1.0	1.0	1.0
POD1	1.12	1.75	2.27	1.2	0.72	1.21	1.94	1.12	1.0	1.12	0.75	1.99
POD3	0.92	2.23	2.01	1.06	0.82	1.38	3.25	1.04	1.0	1.12	0.51	2.24
POD7	1.02	1.77	1.1	1.01	1.0	1.18	3.72	0.99	1.38	1.13	0.83	2.11

	Tnfrsf1a (v) - Panel 1	Bmpr1a (v) - Panel 1	Csf3 (v) - Panel 1	Ephb2 (v) - Panel 1	Fbln5 (v) - Panel 1	Flt1 (v) - Panel 1	Il1b (v) - Panel 1	Jag1 (v) - Panel 1	Ltbp4 (v) - Panel 1	Mmp2 (v) - Panel 1	Pik3ca (v) - Panel 1	Ttf1 (v) - Panel 1
POD0	1.0	1.0	1.0	1.0	1.0	1.0	1.0	1.0	1.0	1.0	1.0	1.0
POD1	1.08	1.07	0.88	1.33	0.86	0.58	0.77	1.09	0.84	1.19	1.14	0.99
POD3	0.98	1.01	0.79	1.68	1.03	0.16	0.57	0.81	0.65	2.66	1.01	0.85
POD7	0.89	1.08	1.06	2.25	1.35	0.34	0.85	0.6	1.09	2.96	1.02	0.98

	Bmpr2 (v) - Panel 1	Ctgf (v) - Panel 1	Ephb4 (v) - Panel 1	Fbn1 (v) - Panel 1	Flt3 (v) - Panel 1	Ilk (v) - Panel 1	Jun (v) - Panel 1	Map2k1 (v) - Panel 1	Mmp9 (v) - Panel 1	Plg (v) - Panel 1	Smad9 (v) - Panel 1	Vegfa (v) - Panel 1
POD0	1.0	1.0	1.0	1.0	1.0	1.0	1.0	1.0	1.0	1.0	1.0	1.0
POD1	1.03	1.08	0.88	1.02	0.86	1.06	1.07	1.41	0.16	0.09	0.58	1.04
POD3	0.88	0.98	0.68	1.35	0.0	1.02	1.08	1.48	0.8	0.19	0.92	0.98
POD7	0.67	1.04	0.8	1.46	0.51	1.02	0.95	1.32	0.36	0.93	0.35	0.98

Generated by Cytobank on 2017-11-03 06:35:31 -0700
