# Supplementary material for: Single-Cell Transcriptional Profiling of Cells Derived From Regenerating Alveolar Ducts
Source: Front Med (Lausanne). 2020 Apr 21;7:112. doi: 10.3389/fmed.2020.00112 (PMC7186418; doi:10.3389/fmed.2020.00112)
Supplement: Supplementary file 3 [file Data_Sheet_3.PDF]

Cytobank

Experiment	single cell histograms
File	Steven's Working Illustration
Created/Updated	2017-10-31 13:20:43 -0700 / 2017-11-03 06:30:02 -0700
Illustration Author	Steven Mentzer
Primary Researcher	Steven Mentzer

Steven's Working Illustration

Histogram Overlay

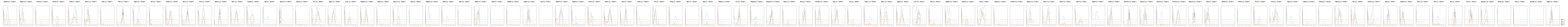

Calculated Fold of Means by First Row using X-Axis channel(s): Panel/Channel Values

	Smad4 (v) - Panel 1	Pdgfra (v) - Panel 1	Acta2 (v) - Panel 1	Casp3 (v) - Panel 1	Ctsk (v) - Panel 1	Ephx1 (v) - Panel 1	Fgf1 (v) - Panel 1	Flt4 (v) - Panel 1	Itgav (v) - Panel 1	Kdr (v) - Panel 1	Mapk1 (v) - Panel 1	Npr1 (v) - Panel 1	Pten (v) - Panel 1	Sphk1 (v) - Panel 1	Akt1 (v) - Panel 1	Casp8 (v) - Panel 1	Egf (v) - Panel 1	Ephx2 (v) - Panel 1
POD0	1.0	1.0	X	1.0	1.0	1.0	X	1.0	1.0	X	1.0	1.0	1.0	1.0	1.0	1.0	1.0	1.0
POD1	0.81	1.07	X	1.79	0.72	0.89	X	1.0	0.33	X	1.34	0.93	1.15	0.94	1.97	1.42	0.0	0.75
POD3	0.61	0.62	X	1.41	1.5	0.81	X	0.96	0.35	X	1.37	0.76	0.75	0.93	1.67	1.38	0.05	0.31
POD7	0.47	1.04	X	0.86	0.88	0.97	X	0.98	0.35	X	1.17	0.97	0.72	0.93	1.43	0.53	0.0	0.46

	Fgf10 (v) - Panel 1	Fn1 (v) - Panel 1	Itgb1 (v) - Panel 1	Kras (v) - Panel 1	Mapk14 (v) - Panel 1	Pdgfa (v) - Panel 1	Ptpn11 (v) - Panel 1	Tgfa (v) - Panel 1	Angpt2 (v) - Panel 1	Cdh5 (v) - Panel 1	Egfr (v) - Panel 1	Erbb2 (v) - Panel 1	Fgf2 (v) - Panel 1	Hbegf (v) - Panel 1	Itgb2 (v) - Panel 1	Lep (v) - Panel 1
POD0	X	1.0	1.0	1.0	1.0	X	1.0	X	1.0	1.0	X	1.0	X	X	1.0	X
POD1	X	1.16	0.92	2.46	0.7	X	0.61	X	0.12	0.68	X	1.7	X	X	1.05	X
POD3	X	1.71	1.01	1.93	0.86	X	0.81	X	0.02	0.1	X	1.16	X	X	1.14	X
POD7	X	1.39	0.96	1.67	0.68	X	0.43	X	0.04	0.15	X	1.63	X	X	1.06	X

	Mapk3 (v) - Panel 1	Pdgfb (v) - Panel 1	S1pr1 (v) - Panel 1	Tgfb1 (v) - Panel 1	Bax (v) - Panel 1	Cela1 (v) - Panel 1	Eln (v) - Panel 1	Ereg (v) - Panel 1	Fgf7 (v) - Panel 1	Hgf (v) - Panel 1	Itgb3 (v) - Panel 1	Lox (v) - Panel 1	Mapk8 (v) - Panel 1	Serpine1 (v) - Panel 1	Timp1 (v) - Panel 1	Bcl2 (v) - Panel 1
POD0	1.0	1.0	1.0	1.0	1.0	1.0	1.0	1.0	X	1.0	X	1.0	1.0	1.0	X	1.0
POD1	1.37	0.81	1.82	0.92	0.82	0.73	1.57	0.3	X	0.21	X	1.18	1.45	1.11	X	1.01
POD3	1.5	0.23	1.03	0.83	0.73	1.02	0.87	0.26	X	0.23	X	1.17	0.78	0.99	X	1.12
POD7	1.15	1.06	2.16	0.84	0.4	0.31	1.55	0.56	X	0.43	X	1.2	0.45	1.06	X	0.99

	Col18a1 (v) - Panel 1	Eng (v) - Panel 1	Fas (v) - Panel 1	Fgfr1 (v) - Panel 1	Hif1a (v) - Panel 1	Itgb5 (v) - Panel 1	Lrp1 (v) - Panel 1	Met (v) - Panel 1	Pdgfrb (v) - Panel 1	Smad2 (v) - Panel 1	Tnf (v) - Panel 1	Bmp4 (v) - Panel 1	Col4a3 (v) - Panel 1	Epha2 (v) - Panel 1	Fbln1 (v) - Panel 1	Fgfr2 (v) - Panel 1
POD0	1.0	1.0	X	1.0	1.0	1.0	1.0	X	1.0	X	1.0	X	X	1.0	1.0	1.0
POD1	1.4	0.99	X	0.72	1.12	0.79	1.17	X	0.6	X	0.95	X	X	1.14	0.98	0.0
POD3	1.21	0.74	X	0.28	1.13	1.25	1.26	X	0.23	X	0.83	X	X	0.64	0.71	0.44
POD7	1.54	0.98	X	0.64	0.92	1.06	1.14	X	0.64	X	0.84	X	X	1.13	1.07	1.05

	Igf1 (v) - Panel 1	Itgb8 (v) - Panel 1	Ltbp2 (v) - Panel 1	Mmp14 (v) - Panel 1	Pecam1 (v) - Panel 1	Smad3 (v) - Panel 1	Tnfrsf1a (v) - Panel 1	Bmpr1a (v) - Panel 1	Csf3 (v) - Panel 1	Ephb2 (v) - Panel 1	Fbln5 (v) - Panel 1	Flt1 (v) - Panel 1	Il1b (v) - Panel 1	Jag1 (v) - Panel 1	Ltbp4 (v) - Panel 1
POD0	X	1.0	1.0	1.0	1.0	1.0	1.0	1.0	1.0	X	X	1.0	1.0	1.0	X
POD1	X	0.0	0.96	1.04	0.9	0.33	1.84	0.96	0.87	X	X	1.06	0.89	0.5	X
POD3	X	0.0	0.93	1.0	0.53	0.06	1.92	1.03	0.61	X	X	1.27	0.92	0.33	X
POD7	X	0.0	1.05	1.08	0.92	0.27	1.19	1.04	0.88	X	X	0.69	1.02	0.0	X

	Mmp2 (v) - Panel 1	Pik3ca (v) - Panel 1	Ttf1 (v) - Panel 1	Bmpr2 (v) - Panel 1	Ctgf (v) - Panel 1	Ephb4 (v) - Panel 1	Fbn1 (v) - Panel 1	Flt3 (v) - Panel 1	Ilk (v) - Panel 1	Jun (v) - Panel 1	Map2k1 (v) - Panel 1	Mmp9 (v) - Panel 1	Plg (v) - Panel 1	Smad9 (v) - Panel 1	Vegfa (v) - Panel 1
POD0	X	1.0	1.0	1.0	1.0	1.0	X	1.0	1.0	1.0	1.0	X	X	X	1.0
POD1	X	1.55	0.85	0.6	1.04	2.08	X	0.45	1.07	1.05	0.96	X	X	X	1.05
POD3	X	1.53	0.92	0.44	1.08	1.9	X	0.17	1.12	1.13	0.8	X	X	X	1.13
POD7	X	1.69	0.87	0.27	1.13	2.32	X	0.72	1.11	1.06	0.69	X	X	X	1.06
