# Supplementary material for: Single-Cell Transcriptional Profiling of Cells Derived From Regenerating Alveolar Ducts
Source: Front Med (Lausanne). 2020 Apr 21;7:112. doi: 10.3389/fmed.2020.00112 (PMC7186418; doi:10.3389/fmed.2020.00112)
Supplement: Supplementary file 6 [file Data_Sheet_6.PDF]

Cytobank

Experiment	single cell histograms
File	Steven's Working Illustration
Created/Updated	2017-10-31 13:20:43 -0700 / 2017-11-03 06:30:02 -0700
Illustration Author	Steven Mentzer
Primary Researcher	Steven Mentzer

Steven's Working Illustration

Histogram Overlay

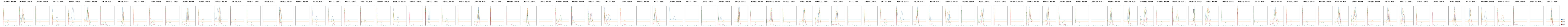

Calculated Fold of Means by First Row using X-Axis channel(s): Panel/Channel Values

	Smad4 (v) - Panel 1	Pdgfra (v) - Panel 1	Acta2 (v) - Panel 1	Casp3 (v) - Panel 1	Ctsk (v) - Panel 1	Ephx1 (v) - Panel 1	Fgf1 (v) - Panel 1	Flt4 (v) - Panel 1	Itgav (v) - Panel 1	Kdr (v) - Panel 1	Mapk1 (v) - Panel 1	Npr1 (v) - Panel 1
POD0	X	1.0	X	1.0	1.0	1.0	X	1.0	X	X	X	1.0
POD1	X	1.5	X	0.5	0.12	1.08	X	1.01	X	X	X	0.98
POD3	X	1.41	X	0.65	0.21	1.24	X	1.01	X	X	X	0.9
POD7	X	1.93	X	0.48	0.1	1.33	X	1.1	X	X	X	1.0

	Pten (v) - Panel 1	Sphk1 (v) - Panel 1	Akt1 (v) - Panel 1	Casp8 (v) - Panel 1	Egf (v) - Panel 1	Ephx2 (v) - Panel 1	Fgf10 (v) - Panel 1	Fn1 (v) - Panel 1	Itgb1 (v) - Panel 1	Kras (v) - Panel 1	Mapk14 (v) - Panel 1	Pdgfa (v) - Panel 1
POD0	X	1.0	X	X	X	1.0	X	1.0	1.0	X	X	X
POD1	X	1.37	X	X	X	1.34	X	0.39	1.16	X	X	X
POD3	X	1.47	X	X	X	0.78	X	0.31	1.85	X	X	X
POD7	X	1.55	X	X	X	0.71	X	0.39	1.01	X	X	X

	Ptpn11 (v) - Panel 1	Tgfa (v) - Panel 1	Angpt2 (v) - Panel 1	Cdh5 (v) - Panel 1	Egfr (v) - Panel 1	Erbb2 (v) - Panel 1	Fgf2 (v) - Panel 1	Hbegf (v) - Panel 1	Itgb2 (v) - Panel 1	Lep (v) - Panel 1	Mapk3 (v) - Panel 1	Pdgfb (v) - Panel 1
POD0	1.0	X	1.0	X	X	1.0	X	X	1.0	X	X	1.0
POD1	0.93	X	0.04	X	X	1.0	X	X	0.87	X	X	3.0
POD3	0.82	X	0.16	X	X	0.97	X	X	0.87	X	X	2.06
POD7	0.18	X	0.04	X	X	1.05	X	X	0.91	X	X	2.15

	S1pr1 (v) - Panel 1	Tgfb1 (v) - Panel 1	Bax (v) - Panel 1	Cela1 (v) - Panel 1	Eln (v) - Panel 1	Ereg (v) - Panel 1	Fgf7 (v) - Panel 1	Hgf (v) - Panel 1	Itgb3 (v) - Panel 1	Lox (v) - Panel 1	Mapk8 (v) - Panel 1	Serpine1 (v) - Panel 1
POD0	1.0	1.0	X	X	1.0	1.0	X	X	1.0	1.0	X	1.0
POD1	1.38	1.36	X	X	1.17	0.0	X	X	0.8	0.93	X	0.85
POD3	1.19	0.46	X	X	1.03	0.0	X	X	1.43	0.98	X	0.86
POD7	2.24	0.94	X	X	1.13	0.0	X	X	0.5	1.01	X	1.07

	Timp1 (v) - Panel 1	Bcl2 (v) - Panel 1	Col18a1 (v) - Panel 1	Eng (v) - Panel 1	Fas (v) - Panel 1	Fgfr1 (v) - Panel 1	Hif1a (v) - Panel 1	Itgb5 (v) - Panel 1	Lrp1 (v) - Panel 1	Met (v) - Panel 1	Pdgfrb (v) - Panel 1	Smad2 (v) - Panel 1
POD0	X	1.0	1.0	1.0	X	X	X	1.0	1.0	1.0	1.0	X
POD1	X	1.0	0.81	0.91	X	X	X	0.23	0.98	0.49	0.66	X
POD3	X	1.08	0.81	0.87	X	X	X	0.13	1.0	0.0	0.34	X
POD7	X	1.0	0.97	0.99	X	X	X	0.04	0.99	0.58	0.41	X

	Tnf (v) - Panel 1	Bmp4 (v) - Panel 1	Col4a3 (v) - Panel 1	Epha2 (v) - Panel 1	Fbln1 (v) - Panel 1	Fgfr2 (v) - Panel 1	Igf1 (v) - Panel 1	Itgb8 (v) - Panel 1	Ltbp2 (v) - Panel 1	Mmp14 (v) - Panel 1	Pecam1 (v) - Panel 1	Smad3 (v) - Panel 1
POD0	1.0	X	X	1.0	1.0	X	X	X	1.0	1.0	1.0	X
POD1	0.85	X	X	1.01	0.99	X	X	X	1.03	0.99	1.0	X
POD3	0.64	X	X	0.93	0.89	X	X	X	1.01	1.11	0.83	X
POD7	0.89	X	X	1.44	1.06	X	X	X	1.13	1.15	1.05	X

	Tnfrsf1a (v) - Panel 1	Bmpr1a (v) - Panel 1	Csf3 (v) - Panel 1	Ephb2 (v) - Panel 1	Fbln5 (v) - Panel 1	Flt1 (v) - Panel 1	Il1b (v) - Panel 1	Jag1 (v) - Panel 1	Ltbp4 (v) - Panel 1	Mmp2 (v) - Panel 1	Pik3ca (v) - Panel 1	Ttf1 (v) - Panel 1
POD0	X	1.0	1.0	X	X	X	1.0	X	X	X	1.0	1.0
POD1	X	0.91	1.04	X	X	X	1.02	X	X	X	0.95	0.87
POD3	X	0.99	0.79	X	X	X	1.06	X	X	X	0.91	0.98
POD7	X	1.01	1.11	X	X	X	0.94	X	X	X	1.1	1.06

	Bmpr2 (v) - Panel 1	Ctgf (v) - Panel 1	Ephb4 (v) - Panel 1	Fbn1 (v) - Panel 1	Flt3 (v) - Panel 1	Ilk (v) - Panel 1	Jun (v) - Panel 1	Map2k1 (v) - Panel 1	Mmp9 (v) - Panel 1	Plg (v) - Panel 1	Smad9 (v) - Panel 1	Vegfa (v) - Panel 1
POD0	X	1.0	1.0	X	X	1.0	1.0	1.0	X	X	X	1.0
POD1	X	0.97	0.93	X	X	0.94	1.06	0.26	X	X	X	0.95
POD3	X	0.96	1.02	X	X	1.0	1.12	0.17	X	X	X	1.01
POD7	X	1.11	1.29	X	X	1.04	1.15	0.12	X	X	X	1.04
