# Supplementary material for: Single-Cell Transcriptional Profiling of Cells Derived From Regenerating Alveolar Ducts
Source: Front Med (Lausanne). 2020 Apr 21;7:112. doi: 10.3389/fmed.2020.00112 (PMC7186418; doi:10.3389/fmed.2020.00112)
Supplement: Supplementary Data — includes single-cell transcriptional data by cluster (Data Sheets 1–3) and post-pneumonectomy day (Data Sheets 4–7). [file Data_Sheet_7.PDF]

Cytobank

Experiment	single cell histograms
File	Steven's Working Illustration
Created/Updated	2017-10-31 13:20:43 -0700 / 2017-11-03 06:30:02 -0700
Illustration Author	Steven Mentzer
Primary Researcher	Steven Mentzer

Steven's Working Illustration

Histogram Overlay

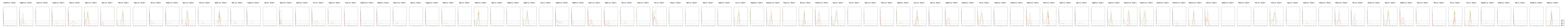

Calculated Fold of Means by First Row using X-Axis channel(s): Panel/Channel Values

	Smad4 (v) - Panel 1	Pdgfra (v) - Panel 1	Acta2 (v) - Panel 1	Casp3 (v) - Panel 1	Ctsk (v) - Panel 1	Ephx1 (v) - Panel 1	Fgf1 (v) - Panel 1	Flt4 (v) - Panel 1	Itgav (v) - Panel 1	Kdr (v) - Panel 1	Mapk1 (v) - Panel 1	Npr1 (v) - Panel 1	Pten (v) - Panel 1	Sphk1 (v) - Panel 1	Akt1 (v) - Panel 1	Casp8 (v) - Panel 1
POD0	X	1.0	1.0	X	X	1.0	X	1.0	X	1.0	X	1.0	X	1.0	X	X
POD1	X	0.5	0.02	X	X	0.74	X	0.9	X	0.31	X	0.94	X	0.93	X	X
POD3	X	0.31	0.02	X	X	0.53	X	0.9	X	0.41	X	0.88	X	0.91	X	X
POD7	X	1.11	0.0	X	X	0.82	X	0.97	X	0.12	X	1.08	X	1.05	X	X

	Egf (v) - Panel 1	Ephx2 (v) - Panel 1	Fgf10 (v) - Panel 1	Fn1 (v) - Panel 1	Itgb1 (v) - Panel 1	Kras (v) - Panel 1	Mapk14 (v) - Panel 1	Pdgfa (v) - Panel 1	Ptpn11 (v) - Panel 1	Tgfa (v) - Panel 1	Angpt2 (v) - Panel 1	Cdh5 (v) - Panel 1	Egfr (v) - Panel 1	Erbb2 (v) - Panel 1	Fgf2 (v) - Panel 1	Hbegf (v) - Panel 1
POD0	X	1.0	X	X	X	X	X	X	X	X	1.0	X	X	1.0	X	X
POD1	X	0.49	X	X	X	X	X	X	X	X	0.06	X	X	1.18	X	X
POD3	X	0.26	X	X	X	X	X	X	X	X	0.02	X	X	0.99	X	X
POD7	X	0.24	X	X	X	X	X	X	X	X	0.03	X	X	1.21	X	X

	Itgb2 (v) - Panel 1	Lep (v) - Panel 1	Mapk3 (v) - Panel 1	Pdgfb (v) - Panel 1	S1pr1 (v) - Panel 1	Tgfb1 (v) - Panel 1	Bax (v) - Panel 1	Cela1 (v) - Panel 1	Eln (v) - Panel 1	Ereg (v) - Panel 1	Fgf7 (v) - Panel 1	Hgf (v) - Panel 1	Itgb3 (v) - Panel 1	Lox (v) - Panel 1	Mapk8 (v) - Panel 1	Serpine1 (v) - Panel 1
POD0	1.0	X	1.0	1.0	X	X	X	X	1.0	X	X	X	X	1.0	X	1.0
POD1	1.16	X	1.05	0.21	X	X	X	X	0.67	X	X	X	X	0.9	X	0.91
POD3	1.05	X	2.04	0.08	X	X	X	X	0.46	X	X	X	X	0.73	X	0.55
POD7	1.18	X	0.51	0.01	X	X	X	X	0.51	X	X	X	X	0.99	X	1.14

	Timp1 (v) - Panel 1	Bcl2 (v) - Panel 1	Col18a1 (v) - Panel 1	Eng (v) - Panel 1	Fas (v) - Panel 1	Fgfr1 (v) - Panel 1	Hif1a (v) - Panel 1	Itgb5 (v) - Panel 1	Lrp1 (v) - Panel 1	Met (v) - Panel 1	Pdgfrb (v) - Panel 1	Smad2 (v) - Panel 1	Tnf (v) - Panel 1	Bmp4 (v) - Panel 1	Col4a3 (v) - Panel 1	Epha2 (v) - Panel 1
POD0	X	1.0	X	1.0	X	X	X	1.0	1.0	X	1.0	X	1.0	X	1.0	X
POD1	X	1.05	X	0.85	X	X	X	0.04	0.98	X	0.48	X	0.87	X	0.0	X
POD3	X	1.23	X	0.67	X	X	X	0.17	1.03	X	0.16	X	0.61	X	0.05	X
POD7	X	1.12	X	0.9	X	X	X	0.0	1.0	X	0.49	X	0.89	X	0.0	X

	Fbln1 (v) - Panel 1	Fgfr2 (v) - Panel 1	Igf1 (v) - Panel 1	Itgb8 (v) - Panel 1	Ltbp2 (v) - Panel 1	Mmp14 (v) - Panel 1	Pecam1 (v) - Panel 1	Smad3 (v) - Panel 1	Tnfrsf1a (v) - Panel 1	Bmpr1a (v) - Panel 1	Csf3 (v) - Panel 1	Ephb2 (v) - Panel 1	Fbln5 (v) - Panel 1	Flt1 (v) - Panel 1	Il1b (v) - Panel 1	Jag1 (v) - Panel 1
POD0	1.0	X	X	X	X	1.0	1.0	X	X	1.0	X	X	X	X	1.0	X
POD1	0.92	X	X	X	X	0.84	0.73	X	X	0.93	X	X	X	X	0.8	X
POD3	0.64	X	X	X	X	0.75	0.53	X	X	0.97	X	X	X	X	0.57	X
POD7	1.12	X	X	X	X	0.96	0.88	X	X	0.97	X	X	X	X	0.83	X

	Ltbp4 (v) - Panel 1	Mmp2 (v) - Panel 1	Pik3ca (v) - Panel 1	Ttf1 (v) - Panel 1	Bmpr2 (v) - Panel 1	Ctgf (v) - Panel 1	Ephb4 (v) - Panel 1	Fbn1 (v) - Panel 1	Flt3 (v) - Panel 1	Ilk (v) - Panel 1	Jun (v) - Panel 1	Map2k1 (v) - Panel 1	Mmp9 (v) - Panel 1	Plg (v) - Panel 1	Smad9 (v) - Panel 1	Vegfa (v) - Panel 1
POD0	X	X	1.0	X	X	1.0	X	X	X	1.0	1.0	X	X	X	X	1.0
POD1	X	X	0.68	X	X	0.95	X	X	X	0.95	0.97	X	X	X	X	1.0
POD3	X	X	0.39	X	X	0.71	X	X	X	0.98	1.07	X	X	X	X	1.13
POD7	X	X	0.76	X	X	1.0	X	X	X	0.99	0.97	X	X	X	X	1.09
